# Adulterations of Food

**Published:** 1881-02

**Authors:** 


ADULTERATIONS OF FOOD.—Supplement No. 11, accompanying the *National Board of Health Bulletin* for January 1st, 1881, is filled with the report of Dr. Charles Smart, U. S. A., giving the results of his investigations concerning the adulterations of articles used for food or in its preparation for use.

The articles examined were teas, coffees, chicory, sugars, syrups, flour, corn-meal, lard, bread, baking-soda, cream of tartar, baking-powder, black pepper, white pepper, allspice, ginger, nutmegs, mace, cloves whole, cloves ground, cinnamon whole, cinnamon ground, cassia, cayenne, mustard, vinegar, pickles and colored confectionery.

The whole number of samples examined was seven hundred and thirteen—three hundred and four of which were obtained from the most reputable dealers, and four hundred and nine from sources that might be regarded as suspicious. Of the former, less than eight per cent. gave evidences of any important adulterations; and of the latter, forty-four per cent. As to the *character* of the adulterations, the report says: "Fortunately, with such exceptions as the alum in bread and the baking material, the sulphate of lime which oftentimes replaces cream of tartar in household baking, the debasement of milk by diluting, and the poisonous pigments used for coloring confectionery, the adulterations cannot be considered as deleterious. They affect the pocket of the individual rather than his health, so that, to use the words of the committee appointed by the National Board of Trade to award prizes for the best draught of an act repressive of adulterations, 'the question of adulteration of food should therefore be considered not so much from a sanitary standpoint as from that of commercial interests; as being in the nature of a fraud in aiding the sale of articles which are not what they are represented to be.'" These results are much more favorable than was anticipated by many, and do not afford any strong indication that special repressive legislation is necessary.

